# Longitudinal prospective anthropometric evaluation in Caucasian prepubertal children with lactose intolerance

**DOI:** 10.3389/fped.2023.1219195

**Published:** 2023-08-21

**Authors:** Mauro Lizzi, Laura Sgrazzutti, Annamaria Porreca, Paola Di Filippo, Chiara Cauzzo, Sabrina Di Pillo, Francesco Chiarelli, Marina Attanasi

**Affiliations:** ^1^ Department of Pediatrics, Pediatric Allergy and Pulmonology Unit, University of Chieti, Chieti, Italy; ^2^ Department of Pediatrics, San Giacomo Hospital, Castelfranco Veneto, Italy; ^3^ Department of Economic Studies, University of Chieti, Chieti, Italy

**Keywords:** children, growth, lactose intolerance, abdominal pain, gastrointestinal disorders, lactose-free diet, milk

## Abstract

**Introduction:**

The health consequences of lactose intolerance remain unclear. We studied the association of lactose intolerance with growth in children.

**Methods:**

In this prospective case–control study, we compared Caucasian prepubertal children with lactose intolerance (LI) [*n* = 30, median age = 7.87 years (3.00–12.75)] to healthy controls [(*n* = 75, median age = 2.25 years (2.00–7.25)]. A lactose tolerance test was performed for lactose intolerance diagnosis. The gastrointestinal symptom score was administered at baseline and after a lactose-free diet for a median period of 9.0 months [range 5%–95% (6.0–24.0)]. The anthropometric parameters were measured at baseline and follow-up. All the anthropometric data were converted into standard deviation scores (SDS). A linear regression model was used to investigate the association of lactose intolerance with growth parameters.

**Results:**

We found no difference in height velocity SDS between the LI and control groups [SDS difference (95% CI): 0.52 (−1.86 to 2.90)]. In addition, we found a significant reduction in the clinical score of the LI group after a lactose-free diet [median (5%–95%): 7.5 (4.0–15.0) and 3 (0.0–8.0); *p-*value <0.001].

**Conclusions:**

The LI group exhibited no difference in height velocity compared with the control group. Nonetheless, due to the small sample size, the results on the anthropometric profile of the LI group require careful interpretation. More large-scale studies in the pediatric population are required to better understand the association of LI with anthropometric and metabolic profiles.

## 1. Introduction

Pediatricians should maintain awareness of the beneﬁts and controversies related to the consumption of dietary milk products and milk-based infant formula. The American Academy of Pediatrics recommends the consumption of dairy products as an important source of calcium (Ca) for bone mineral health and proper growth in children and adolescents (1). Primary lactose intolerance (LI) affects approximately 70% of the world's population, with the percentage varying according to ethnicity and dairy product use (2). LI is caused by the inability to digest and absorb dietary lactose, a disaccharide that must be hydrolyzed into its component simple sugars to be absorbed in the intestinal mucosa. This hydrolysis is accomplished by lactase, an essential enzyme produced in the intestinal mucosa of all young mammals (3). Bacterial fermentation of the non-digested lactose leads to the production of short-chain fatty acids and gas (4). The gastrointestinal symptoms, such as bloating, abdominal pain, flatulence, diarrhea, and rarely nausea and vomiting, occur depending on the degree of elevation of gastric gas (4). The LI subjects are usually prescribed with a lactose-free diet to avoid symptoms related to their syndrome (4). Other nutrients such as protein make dairy products an important source of nutrition for growing children (5). A controversial area in LI research is whether the lactose-free diet may lead to lower protein and calcium intake, which consequently leads to poor growth, short stature, and low bone mineral density (6). Some investigators have suggested that an adequate calcium intake during the growth period may be critical for reaching optimal bone growth in childhood (7), and others have provided evidence on the short stature of LI children, those with milk allergy, or those on milk-elimination diets (8, 9). A recent analysis reports that the biological sequelae of routine milk consumption, such as statural growth, remain unclear (10). Furthermore, no large-scale randomized trial study in prepubertal children has been yet conducted to accurately assess this hypothesis. This study aims to evaluate the associations of lactose intolerance with growth velocity in children having a lactose-free diet.

## 2. Materials and methods

### 2.1. Study design and subjects

We conducted a longitudinal prospective study from January 2020 to January 2023 at the Department of Pediatrics, University of Chieti, Italy. We enrolled 73 prepubertal children who had suggestive LI abdominal symptoms (abdominal pain, diarrhea, nausea, ﬂatulence, and/or bloating after ingesting lactose or lactose-containing food) and who attended the Pediatric Allergy and Pulmonology Unit. A primary care pediatrician recruited 107 children without any disease affecting growth and pubertal development for the control group. The exclusion criteria were severe hypothyroidism, pseudohypoparathyroidism, Cushing's syndrome, diabetes mellitus, nephrotic syndrome, chronic renal disease, chronic inflammatory bowel disorders, celiac disease, infectious enteritis, immunological, hematological, and neoplastic diseases, calcium and/or vitamin D metabolism abnormalities. The subjects receiving calcium, vitamin D or multivitamin supplementation, and antibiotics in the last 2 weeks were also excluded. In addition, children born with low birth weight (<2,500 g) and a history of significant weight loss or gain (>10% of body weight change in the previous 6 months) were excluded. We collected data on past medical history, physical examination, and laboratory tests for fasting blood glucose, cortisol, urinalysis, comprehensive metabolic panel, serum creatinine, and thyroid function. We had no access to information that could identify individual participants during or after data collection. Written informed consent was obtained from parents or legal representatives of all children. The study was approved by the local Ethical Committee of the University of Chieti (INT-LAT, protocol number 8536) and was conducted in compliance with the Declaration of Helsinki.

### 2.2. Anthropometric measurements

In our clinical practice, a pediatric endocrinologist performed the anthropometric measurements and pubertal staging assessment on all outpatients. Tanner's criteria were used to determine the pubertal onset based on the direct evaluation of breast development in girls, testicular size in boys, and pubic hair development in both sexes (11, 12).

The height of the subjects, without wearing shoes, was measured three times with a Harpenden stadiometer (Holtain, Wales, UK) to the nearest 0.1 cm. In addition, the weight of the subjects, wearing light clothing, was measured to the nearest 0.1 kg with a calibrated scale (Salus, Inc., Milan, Italy). Each subject stood straight with feet placed together and ﬂat on the ground; heels, buttocks, and scapulae against the vertical backboard; arm loose and relaxed with the palms facing medially; and the head in the Frankfurt plane position. The scale and stadiometer were calibrated before each study visit. All height and weight data were adjusted for age and gender by calculating the standard deviation scores (SDS) at each visit (pre- and post-follow-up visits), based on the Italian population reference data (13). The body mass index (BMI) was calculated using the formula weight/height^2^ (kg/m^2^) and expressed as SDS for age and gender. In addition, the height and weight velocity SDS was calculated with the following formula: [(height or weight SDS at follow-up − height or weight SDS at baseline)/time (months)] × 12.

Data on parents’ height were collected by phone call and direct measurement in the clinic during the child's visit. The target height for children was calculated using the formulae, i.e., (paternal height + maternal height + 13)/2 cm and (paternal height + maternal height − 13)/2 cm, for boys and girls, respectively (14). The anthropometric data were converted into SDS.

### 2.3. Lactose tolerance test

There is no gold standard available for LI diagnosis. The lactose tolerance test (LTT) has a reasonable sensitivity (0.94; CI: 0.90–0.97) and specificity (0.90; CI: 0.84–0.95), and a good agreement between the lactose hydrogen breath test (H2-BT) and LTT (15) was found. The test was performed at the hospital in the morning. Informed consent was taken from the participants. The patients were put on a low-fiber diet for 48 h before the test and permitted to eat and drink during the last 12 h before the test, brush their teeth, or take any medications. Physical activity, except for the limited activity to reach the hospital, was also prohibited in the morning before the test. After an overnight fast, the patients were given 2 g of lactose/kg body weight up to a maximum of 50 g, which is dissolved in water, and encouraged to finish the drink within 5 min. To test the plasma glucose concentration, capillary blood samples were taken from each patient fasting at −5, 0, 15, 30, 45, and 60 min after lactose ingestion. The average of the −5 and 0 min determinations was used as the pre-challenge glucose concentration (16). Glucose was measured in whole blood using a plasma-calibrated portable glucose meter (Contour XT Blood Glucose Meter, Basel, Switzerland). The cutoff value for defining LI was a glucose concentration of <20 mg/dl above the baseline (17). The symptoms that developed in the patients within 24 h after lactose intake were recorded by phone call. They answered three main questions: (1) when did the symptoms start (during the test or 0–2, 2–4, 4–8, >8 h after the test); (2) what type of symptoms have you complained of (abdominal pain/discomfort, bloating, diarrhea, or constipation); and (3) how long did the symptoms last (0–2, 2–4, 4–8, >8 h)?

### 2.4. Questionnaire

The subjects were asked to rate the presence and severity of gastrointestinal symptoms before and after having a lactose-free diet (18). Abdominal pain, abdominal distention, abdominal bloating, and flatulence (gas) were scored as follows: 0 = no symptoms, 1 = mild symptoms, 2 = moderate symptoms, and 3 = severe symptoms. Bowel movements were scored as 0 = none, 1 = 1 stool, and 2 = ≥2 stools. Stool consistency was scored as 0 = normal or firm, 1 = loose, and 2 = watery. The number of points for each category was totaled to determine the overall degree of symptoms. For the analysis, symptom scores from 0 to 6 were considered as no/mild symptoms, whereas symptom scores ≥7 were considered as moderate/severe symptoms.

### 2.5. Statistical analysis

In our study, all the anthropometric parameters (height, weight, BMI, and growth velocity) were adjusted for age and sex and converted into SDS using the Italian population growth charts (13), at baseline and follow-up. The data showing a non-normal distribution were expressed in median, with a range of 5%–95%.

The chi-squared test was used for categorical variables (sex, mode of delivery, smoke exposition, and family history of allergy), whereas the Wilcoxon signed-rank test and Mann–Whitney *U*-test were used for numerical variables (i.e., age, height, weight, BMI). A multivariable linear regression model was used to investigate the association of lactose intolerance with the height and weight velocity SDS, adjusted for sex, age at enrollment, genetic target, BMI SDS, and months of having a lactose-free diet. The confounders were initially selected from the literature and subsequently tested for their association with both the determinant and outcome or the change of the unadjusted effect estimates of ≥10% when added to the univariate model (19, 20). All measurements of association were presented as SDS differences with corresponding 95% confidence intervals. The statistical significance level was *p* < 0.05. The SPSS version 25.0 for Windows (IBM, Armonk, NY, USA) and STATA/IC 15.1 (StataCorp, 2017. Stata Statistical Software: Release 15. StataCorp LLC. College Station, TX, USA) were used to perform statistical analyses.

## 3. Results

**Figure 1:**
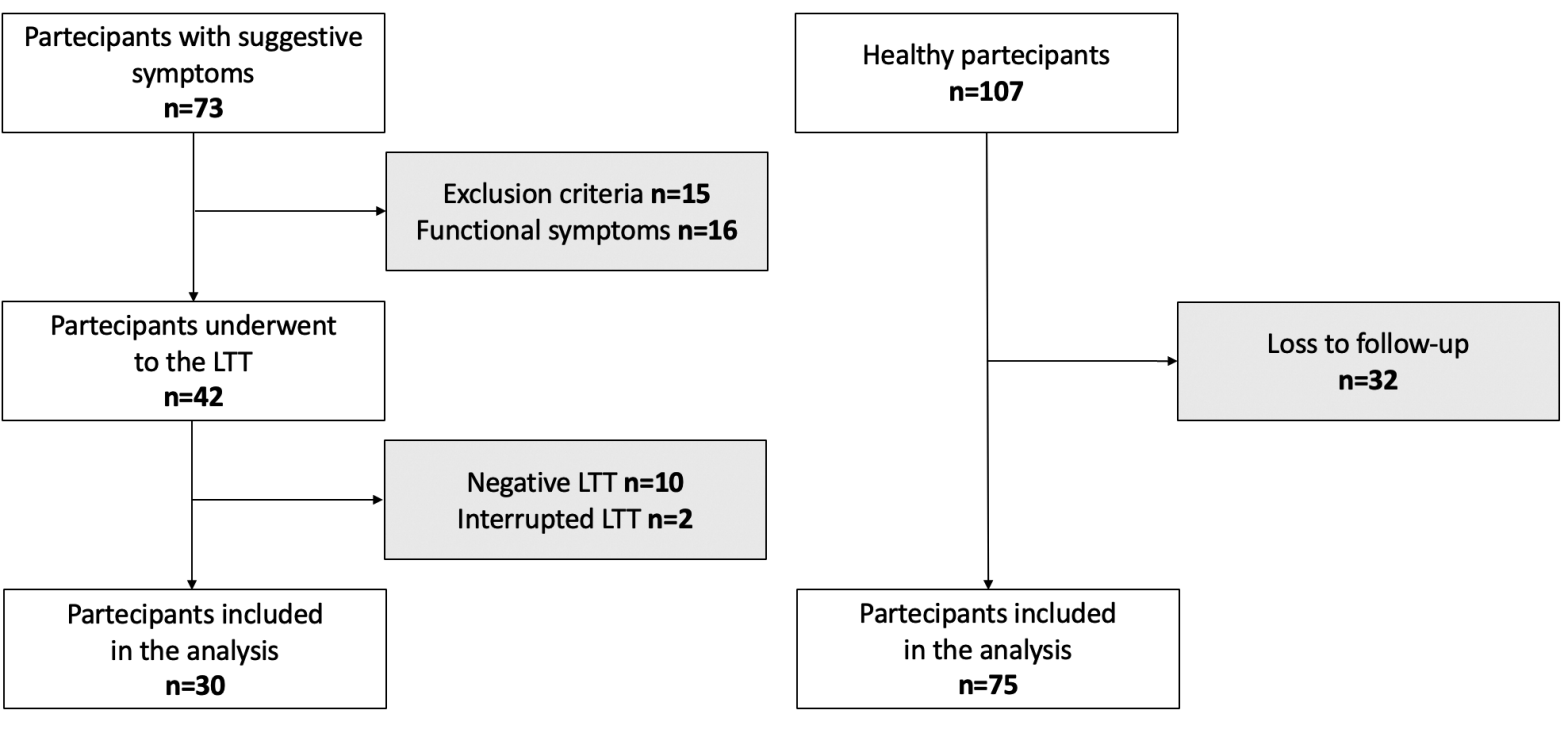
Flowchart of the study. LTT, lactose tolerance test.

The flowchart of the study is shown in Figure 1, and the birth and maternal characteristics are listed in Table 1.

**Table 1:** Birth and paternal characteristics of the study population.

	Control group	LI group	*p*
	*n* = 75	*n* = 30	
Sex (%)			0.577*
Male	42 (56.0)	15 (50.0)	
Female	33 (44.0)	15 (50.0)	
Birth characteristics			
Weight at birth (kg)	3.25 (2.53–4.50)	3.37 (2.58–4.44)	0.180
Mode of delivery (%)			0.121*
Spontaneous	30 (40.0)	17 (56.7)	
Cesarian section	45 (60.0)	13 (43.3)	
Gestational age (weeks)	39.0 (37.0–41.0)	39.0 (37.0–42.0)	0.313
Smoke exposition, yes (%)	30 (40.0)	7 (23.3)	0.106*
Parental characteristics			
Allergy family history, yes (%)	23 (30.7)	23 (76.7)	**<0.001** *
Mother’s height (cm)	163.0 (148.0–177.0)	165.5 (150.0–180.0)	**0** **.** **030**
Father's height (cm)	175.0 (165.0–195.0)	175.0 (166.0–193.0)	0.429
Genetic target (cm)	172.0 (153.5–182.5)	171.0 (156.5–192.5)	0.473

Data are expressed as medians and range, percentages, and absolute numbers.

Bold indicates *p-*value <0.05.

*p*-value obtained from the Mann–Whitney *U*-test. *p-value obtained from the χ^2^-squared test.

In this study, we compare the LI group comprising 30 Caucasian prepubertal LI children to the control group comprising 75 healthy subjects. We found a difference in family history of allergy between the LI and control groups (76.7% vs. 30.7%; *p* < 0.001). The anthropometric measurements at baseline are listed in Table 2.

**Table 2:** Anthropometric characteristics of participants at baseline.

	Control group	LI group	*p*
	*n* = 75	*n* = 30	
Anthropometric parameters at baseline			
Age (years)	2.25 (2.00–7.25)	7.87 (3.00–12.75)	**<0** **.** **001** *
Height SDS	−0.20 (0.9)	0.23 (1.4)	0.067
Weight SDS	−0.26 (1.0)	0.38 (1.1)	**0** **.** **005**
BMI SDS	−0.23 (1.0)	0.39 (1.1)	**0** **.** **009**

Data are expressed as means and standard deviation, medians, and range.

Bold indicates *p*-value <0.05. *p*-value obtained from the unpaired *t*-test.

* *p*-value obtained from the Mann–Whitney *U*-test.

**Figure 2:**
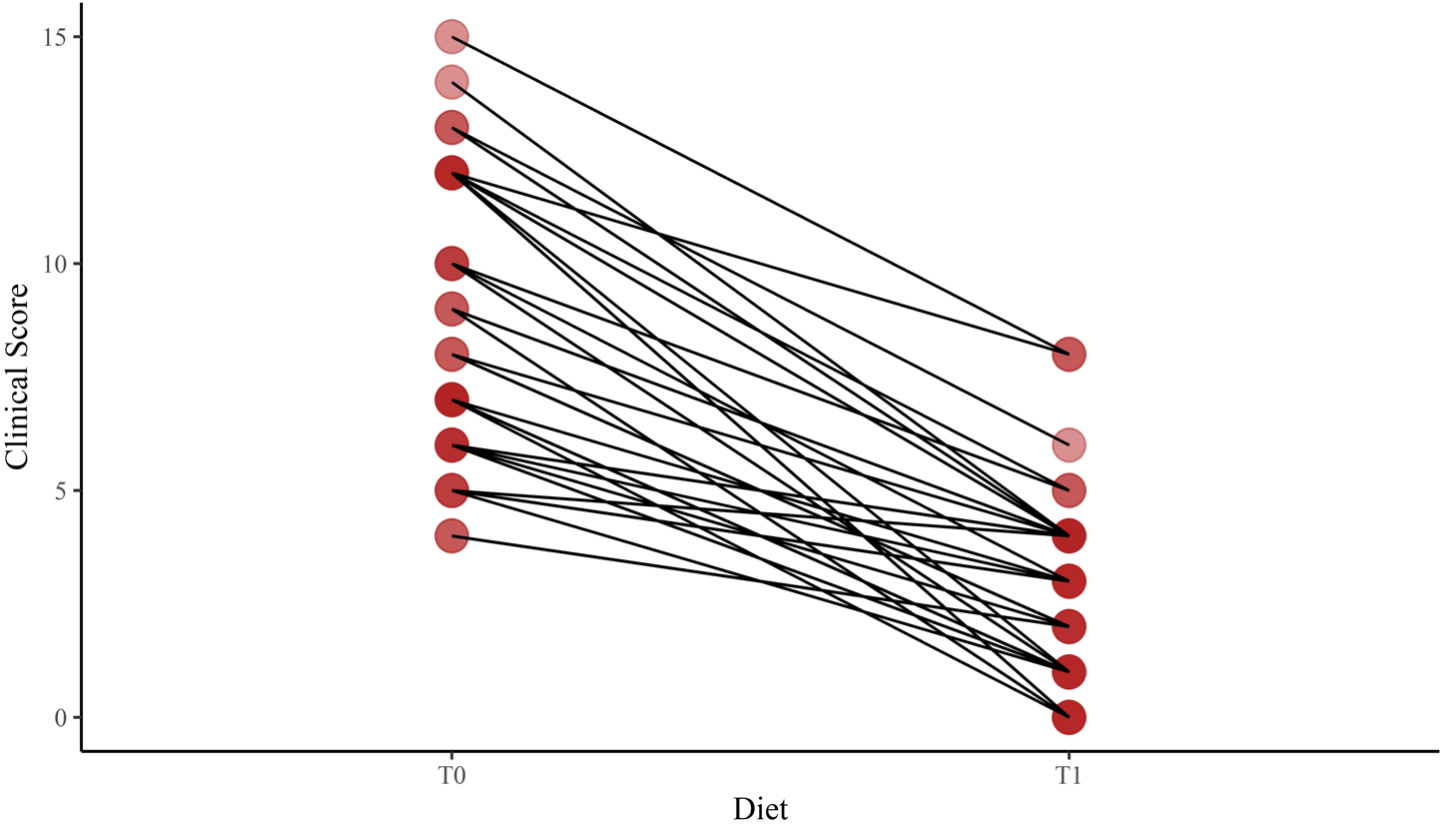
Change of the clinical score after a lactose-free diet in lactose-intolerant children. T0, baseline; T1, follow-up visit after a lactose-free diet.

We found a significant difference in age at enrollment between the LI and control groups [median (5%–95%) 7.87 (3.00–12.75) vs. 2.25 (2.00–7.25); *p* < 0.001]. However, the auxological parameters were expressed in SDS. No difference in the mean height SDS was found between the LI and control groups [mean (SD) 0.23 (1.4) vs. −0.20 (0.9); *p* = 0.067]. The mean weight and BMI SDS were higher in the LI group than in the control group [0.38 (1.1) vs. −0.26 (1.0); *p* = 0.005; 0.39 (1.1) vs. −0.23 (1.0); *p* = 0.009; respectively]. In addition, no difference in target height was found between the LI and control groups [171.0 (156.5–192.5) vs. 172.0 (153.5–182.5); *p* = 0.473]. The gastrointestinal symptom scores were administered at baseline and after having a lactose-free diet with a median period of 9.0 months [5%–95% (6.0–24.0)]. We found a significant reduction in the clinical score of the LI group after having a lactose-free diet [median (5%–95% range) 7.5 (4.0–15.0) and 3 (0.0–8.0); *p-*value < 0.001] (Figure 2).

The anthropometric parameters during the follow-up visit are listed in Table 3.

**Table 3:** Anthropometric characteristics of participants at follow-up.

	Control group	LI group	*p*
	*n* = 75	*n* = 30	
Anthropometric parameters at follow-up			
Age (years)	4.25 (2.75–9.25)	8.87 (3.50–13.50)	**<0** **.** **001** *
Height SDS	−0.05 (1.4)	0.17 (1.4)	0.471
Weight SDS	−1.18 (1.0)	0.38 (1.1)	**0** **.** **014**
BMI SDS	−0.25 (1.1)	0.41 (1.1)	**0** **.** **007**
Height velocity SDS	0.93 (2.0)	−0.52 (3.8)	**0** **.** **012**
Weight velocity SDS	0.08 (0.4)	0.11 (0.6)	0.822

Data are expressed as means and standard deviation, medians, and range.

Bold indicates *p-*value < 0.05. *p*-value obtained from the unpaired *t*-test.

* *p*-value obtained from the Mann–Whitney *U*-test.

During follow-up, the LI group showed higher mean weight and BMI SDS than the control group [0.38 (1.1) vs. −1.18 (1.0), *p* = 0.014; 0.41 (1.1) vs. −0.25 (1.1), *p* = 0.007, respectively]. Notably, the height velocity SDS was lower in the LI group than the control group [−0.52 (3.8) vs. 0.93 (2.0); *p* = 0.012)], although no difference in weight velocity SDS was found between the LI and control groups [0.11 (0.6) vs. 0.08 (0.4); *p* = 0.822].

In the crude model, the LI group showed lower height velocity SDS than the control group [SDS difference (95% CI) −1.45 (−2.57 to −0.32)]. However, after some adjustments, no difference in height velocity SDS was found between the LI and control groups [SDS difference (95% CI) 0.52 (−1.86 to 2.90)].

## 4. Discussion

To the best of our knowledge, this is the first study with a longitudinal assessment of the anthropometric parameters in LI children. It is presumed that LI-associated gastrointestinal symptoms lead to dietary dairy avoidance and to nutritional imbalances associated with lower anthropometric indexes (21). In our study, 70% of the LI group had a moderate-to-severe symptom severity score at enrollment. Since the symptom score at the follow-up was significantly lower than the score at the beginning, we speculated that the LI group strongly limited or avoided dairy products from the diet. However, no association of LI with height velocity SDS was found. In addition, no difference in weight velocity SDS was found between the LI and control groups.

Milk contains essential nutrients and anabolic hormones that nourish and support the growth of young mammals (22). To date, several studies have shown that milk consumption can increase longitudinal growth and height (23, 24). It is unclear which component has the greatest growth-promoting effect. Branched-chain amino acids such as leucine, isoleucine, and valine, contained in cow's milk, increase plasma concentration of insulin-like growth factor 1 (IGF-1), mediating the growth hormone action (25). In addition, leucine influences cell replication through the mammalian target of the rapamycin (mTOR) pathway (26). There is a progressive reduction in lactase levels in children from many ethnic groups after they are weaned [lactase non-persistence (LNP)]. LNP affects approximately 70% of the world's population and is the physiological basis for primary LI (27). Several single nucleotide polymorphisms in the lactase gene promoter region on chromosome 2 are associated with lactase persistence beyond infancy (28). We did not carry out a genetic investigation in our study population. However, we hypothesized that our participants were not affected by developmental lactose intolerance, a characteristic form of a preterm infant, nor by congenital lactase deficiency (alactasia), which is a rare and severe autosomal recessive disorder of the newborn infant (29). In addition, the factors (infection, inflammation, or trauma) that could lead to secondary LI were considered among the exclusion criteria. Therefore, the age and clinical characteristics suggested that children might be carriers of primary LI, the most common cause of LI (30). Currently, no data on the long-term effects of a lactose-free diet in children were reported.

We investigated the LI effect on statural growth during childhood. Indeed, the LI group showed no difference in height velocity SDS compared with the control group. In a cross-sectional study, Setty-Shah et al. (31) found no significant difference in both 25-hydroxyvitamin D levels and height z-score between the LI and control groups. A 21-year longitudinal cohort study of 2,265 Finnish children and adolescents found no association of the lactase-phlorizin hydrolase C/C-13910 genotype with the mean growth speed and final mean body height for both sexes. However, the C/C-13910 genotype had an important effect on dairy consumption and calcium intake, which lasted from childhood to young adulthood (32). In a recent cross-sectional study including 87 children aged 6–17 years, Pienar et al. (33) found no association of gene polymorphisms for primary LI with anthropometric parameters (height, weight, BMI) and metabolic profile [fasting blood glucose, triglycerides (TG), HDL cholesterol]. In contrast to our findings, Stallings et al. (7) found that LI children were significantly shorter compared to unaffected peers. Moreover, Black et al. (6) found that prepubertal children with a history of milk avoidance >4 months from various causes had lower height z-scores compared to the control subjects. Few studies on the relationship between milk consumption and body weight in children were published, and most of them were subject to confounding and reverse causation. A randomized controlled trial including 98 overweight and obese Chilean children aged 8–10 years examined the effects of delivering milk instead of sugar-sweetened beverages on body composition and found no changes in body weight or BMI (34). In a prospective study including 2,245 children, Noel et al. (35) also found no association between milk intake and percentage of body fat. To date, studies on the relationship between LI and weight are also scarce. Setty-Shah et al. (31) found that children with LI had significantly reduced weight and BMI z-scores. Lehtimäki et al. (36) evaluated the metabolic profile of 2,109 young Finns and found no significant difference between the subjects with and subjects without gene polymorphisms for primary LI. Importantly, Almon et al. (20) found that lactose tolerance in adolescents led to higher BMIs. In addition, using larger cohorts, Kettunen et al. (37) and Lamri et al. (38) found that lactose tolerance in adulthood was associated with higher BMIs.

However, some methodological limitations need to be discussed. First, both groups having different ages could have affected our findings, although we adjusted our linear regression model for age to evaluate the independent effect of lactose intolerance on weight growth. Second, we might not have had information on all possible confounders, such as vitamin D basal level and physical activity, which could potentially have an effect given the importance of those two factors in bone growth. However, the limitation of not including a physical activity recording could be mitigated by the fact that there is no clear evidence in the literature on the effect of physical exercise on a child's linear growth (39). Third, we have not considered the presence of lactose-free dairy products in the patients’ diet. For LI people it is nowadays not necessary to completely avoid dairy product intake. Lactose-free dairy products are obtained by the hydrolysis of lactose with neutral lactases or acid lactases (5). The wide availability of lactose-free products could provide the essential nutrients present in regular dairy products, such as calcium, vitamins, and milk proteins, potentially reducing dietary differences (40). In addition, selection bias was also possible in our study because both the case and control groups were drawn from different populations, i.e., the case group from a community hospital and the control group from a general population, leading to an alteration of the target population. Lastly, the study was performed in a high-risk population, comprising children with LI symptoms who attended a third specialized center reducing generalizability to the general population.

The major strength of our study was the longitudinal analysis of the anthropometric parameters with the investigation of height and weight velocity SDS. We also adjusted for the confounders which are important in the association of lactose intolerance with growth measurements. In addition, we enrolled only prepubertal children avoiding the potential effect of the hormonal change of puberty on growth.

In conclusion, LI children showed no difference in height velocity compared with controls. Still, due to the small sample and different ages of the case and control groups, the results concerning the anthropometric profile of LI children require careful interpretation. More large-scale studies in the pediatric population are required to better understand the association of LI with anthropometric and metabolic profiles.

However, pediatricians should carefully assess the growth in LI children in treatment with a lactose-free diet, providing correct information on the dietary regimen to be followed and discouraging the elimination of dairy products from the diet in favor of more caloric foods.

## Data Availability

The raw data supporting the conclusions of this article will be made available by the authors, without undue reservation.
